# Evidence-based support for the use of proton pump inhibitors in cancer therapy

**DOI:** 10.1186/s12967-015-0735-2

**Published:** 2015-11-24

**Authors:** Stefano Fais

**Affiliations:** Department of Therapeutic Research and Medicines Evaluation, National Institute of Health (Istituto Superiore di Sanità), Viale Regina Elena 299, 00161 Rome, Italy

## Abstract

‘We can only cure what we can understand first’, said Otto H. Warburg, the 1931 Nobel laureate for his discovery on tumor metabolism. Unfortunately, we still don’t know too much the mechanisms underlying of cancer development and progression. One of the unsolved mystery includes the strategies that cancer cells adopt to cope with an adverse microenvironment. However, we knew, from the Warburg’s discovery, that through their metabolism based on sugar fermentation, cancer cells acidify their microenvironment and this progressive acidification induces a selective pressure, leading to development of very malignant cells entirely armed to survive in the hostile microenvironment generated by their own metabolism. One of the most mechanism to survive to the acidic tumor microenvironment are proton exchangers not allowing intracellular acidification through a continuous elimination of H^+^ either outside the cells or within the internal vacuoles. This article wants to comment a translational process through which from the preclinical demonstration that a class of proton pump inhibitors (PPI) exploited worldwide for peptic ulcer treatment and gastroprotection are indeed chemosensitizers as well, we have got to the clinical proof of concept that PPI may well be included in new anti-cancer strategies, and with a solid background and rationale.

## Introduction

While the research in cancer with the purpose of discovering new more effective and less toxic therapies is burning, fuelled by excellent ideas, it is at least astonishing to read what is published in recent report by Globocan. This document has teached us that in 2012 approximately 32.6 million people were living with cancer (within 5 years of diagnosis), 14.1 million were adults newly diagnosed and 8.2 million of these cancer patients died. (Globocan, http://globocan.iarc.fr/Pages/fact_sheets_cancer.aspx) [1]. These data should oblige us to think that there is a urgent need for strategies that will help the humanity to face off in short time with this very aggressive problem; with the aim of both preventing and treating cancer with safe and effective new treatments resulting in durable disease remission and increased overall survival. The most recent approaches in the war against cancer were based on the success of antibiotics that was inspired by the magic bullets’ Paul Ehrlich’s principle, introduced more than 100 years ago. The successful use of antibiotics against infectious agents supported the use of the same approach against malignant tumours: to set up new drugs that selectively target and kill tumour cells [2]. After so many years we are still waiting for the magic bullet against malignant tumours. New approaches are now being proposed, such as developing therapeutic strategies aimed at controlling cancer rather than trying to cure it [3]. However, it is also possible to approach new anticancer therapies by trying to understand the mechanisms through which cancer cells avoid growth control. It is possible that cancers also use the same mechanisms to overcome the cytotoxic effect of chemotherapeutic agents, which very often induce more adverse side effects than real benefits. Moreover, despite the use of multiple drug combination protocols and the development of novel targeted anticancer strategies, chemoresistance remains a big problem in cancer treatment [4]. Further understanding on the “life style” of malignant tumours is required. Tumor metabolism and microenvironmental acidity are both involved in tumor resistance to therapies and in allowing growth and progression against a poorly armed body reaction. One of the best defined cascade of events occurring into the tumor microenvironment is the so called “Warburg effect” [5], as represented by an aberrant metabolic pathway of tumors, initially triggered by the hypoxic conditions that selects cells able to survive at low oxygen levels by fermentating sugars and releasing lactate, thus leading to extracellular acidification [6]. Within the tumor mass the rapid turnover of aberrantly dividing cancer cells, implying peculiar glucose utilization, amino acid metabolism and ATP hydrolysis, leads to production and release of large amounts of protons into the extracellular compartment, [7–10]. One intriguing hypothesis is that the highly competitive microenvironment, secondary to tumor proliferation and metabolism, selects the cells best adapted to survive in these hostile conditions. Uncontrolled tumor cell proliferation, acid production (secondary to tumor metabolism) and tissue hypoxia (secondary to low blood supply), all contribute to generate a highly hostile tumor microenvironment with conditions that are unsuitable for most cells. In order to thrive in such an unfavorable microenvironment, tumor cells must develop systems to actively extrude excess protons [9, 11]. These mechanisms include V-ATPase, Na^+^/H^+^ exchanger (NHE), monocarboxylate transporters (MCTs) and carbonic anhydrase 9 [9]. We have performed a series of pre-clinical and clinical studies highly supporting the use of a class of proton pump inhibitors (PPI) currently used for the treatment of peptic disease and as gastroprotectors, and including omeprazole, esomeprazole, lansoprazole, pantoprazole and rabeprazole, in the treatment of cancer patients as well [12, 13]. This commentary will introduce the readers to this discovery in cancer research from the pre-clinical studies [14–18] to the four clinical studies performed in either human [19, 20] and domestic animal [21, 22] patients.

## The novelty and the future

The peculiar anaerobic or aerobic metabolism of glucose by cancer cells leads to the accumulation of acid byproducts resulting in an acid milieu that strongly affects tumor cells and their host [7, 8, 23, 24] Low extracellular/intratumoral pH is a major cause of tumor unresponsiveness to the vast majority of cytotoxic drugs, mostly because the H^+^ rich tumor microenvironment leads to protonation of the chemotherapeutic agent causing both its neutralization outside the cells and prevention of reaching its intracellular targets [7, 8, 23, 24]. The prime cause of tumor microenvironment acidification is secondary to the byproducts of tumor metabolism, namely protons, coupled with reduced perfusion. However, this condition progressively selects cells adapted to survive in the acidic extracellular tumor microenvironment, which is due to overexpression and activation of membrane-bound pH-regulating systems that contribute to prevent intracellular acidification. Among them, vacuolar-type H^+^ ATPases seem to be involved in the acidification of tumor microenvironment [9, 23, 24]. Vacuolar H^+^ ATPase (V-ATPase) is a complex multisubunit protein devoted to the transport of protons from the cytoplasm towards intracellular compartments and from inside to outside of the cell through the cytoplasmic membrane [10]. V-ATPases are made of a transmembrane subunit, named V0 complex, devoted to proton transfer and a cytoplasmic portion, named V1 complex, that provides the necessary energy for proton translocation [25]. Because of its role in the regulation of cellular pH homeostasis, V-ATPase is involved in multiple cellular functions including endocytosis and activation of proteases [25], angiogenesis [26], autophagy [27] and amino acids sensing via interaction with mTOR [28]. Tumor cells located at the margin of neoplastic masses are often away from newly formed blood vessels, receiving and inadequate supply of oxygen and nutrients. Such cells survive and adapt to a highly selective environment characterized by hypoxic and acidic conditions caused by increased glycolysis and reduced tissue perfusion [29]. Augmented expression of V-ATPase is considered to be a well-designed compensatory mechanism that in fact confers survival and growth advantages to cancer cells [29–33]. Among its activities, V-ATPase contributes to lower extracellular pH (pHe) thus activating extracellular metalloproteinases that promote tumor cell survival, motility and invasion, resulting in enhanced malignancy ability. There is a bulk of evidence that points out the role of V-ATPase in tumor invasion and multidrug resistance in breast cancer [34–37], oral squamous cell carcinoma [38–40], esophageal carcinoma [41], hepatocellular and pancreatic carcinoma [42, 43], lung carcinoma [44], sarcoma [45, 46] and solid tumors in general [47]. Consequently, inhibition of V-ATPase has become a fascinating and promising strategy to counteract proton metabolism in cancer, which has been investigated in vitro and in vivo, in both preclinical and clinical settings.

From the first preclinical evidences showing that PPI may work either as chemosensitizing agent [14, 17] or highly cytotoxic anti-tumor agents [15, 16, 18], to the clinical evidences that PPI chemosensitize either human [19, 20] or pets tumor patients [21, 22], the proof of principle is becoming solid and convincing. PPI were able to chemosensitize human tumor cells of different histologies, through a normalization of extracellular pH, both in vitro and in vivo [14–17]. Actually, one common feature of tumors is that they are acidic [7, 8] and the way tumors become acidic is not entirely made clear. However, an interesting hypothesis is that during the primary tumor growth malignant cells develop what is also called “Warburg Effect”, that is the ability of cancer cells to fermentate sugars with lactate production, independently on the oxygen levels within the tumor mass [5, 6]. The condition of H^+^ accumulation within the tumor tissues progressively selects tumor cells armed to survive in this hostile microenvironment [8]. One of the most recognized mechanism allowing cancer cells to survive in the acidic milieu are a series of proton exchangers [9], that help the tumors cells in avoiding intracellular acidification. Between these exchangers there are some proton pumps, such as vacuolar ATPases, that are extremely active in tumor cells by pumping H^+^ both from the cytosol to internal vacuoles and from the plasma membrane to the extracellular microenvironment [9, 10]. Thus, the first idea was to inhibit V-ATPases in order to deprive cancer cells of this mechanism, but direct inhibition of these proton pumps was toxic, being V-ATPases ubiquitary into the body [10, 25]. We focused our attention on a family of proton pump inhibitors (PPI) that are used worldwide as very potent antiacidic drugs against peptic diseases or as gastro-protectors (i.e. omeprazole, esomeprazole, lansoprazole, pantoprazole and rabeprazole) [48], that did not show relevant systemic toxicity, even in prolonged treatments and at very high dosages, as in patients with Zollinger and Hellison syndrome, but also in other disease conditions [49]. PPI specifically target gastric H^+^/K^+^ATPases, but VATPases as well [9, 10, 48]. PPI are prodrugs needing protonation in acidic milieu to be transformed into the active molecule, while chemical drugs are mostly weak bases, undergoing neutralization outside the tumor cells by protonation [50]. Thus, while acidity represents a potent mechanism of tumor resistance to drugs, PPI exploit tumor acidity to become functional [50–52].We thus started with a series of preclinical investigations showing that PPI sensitize tumor cells and tumors to the action of chemotherapeutics [14, 17]. However, we also showed that PPI per se exert a potent antitumor activity, through an in vivo modulation of tumor pH [15, 16]. Lastly we showed that acidity represents a potent mechanism of tumor immune escape and PPI increase the immune reaction against tumors [53]. These preclinical data represented the background for a series of clinical studies aimed at supporting the use of PPI as chemosensitizers. Up to now the results of two clinical trials in humans are published in either osteosarcomas or metastatic breast cancer patients (MBC) [19, 20]. The results showed that pre-treatment with PPI increased the effectiveness of neoadjuvant chemotherapy in osteosarcoma patients, particularly in the chondroblastic variant [19] and the time to progression (TTP) or overall survival (OS) in MBC patients maintained under PPI treatment for one year after the stop of chemotherapy [20]. Moreover, two clinical studies in companion animals with spontaneous tumors, highly supported the efficacy of PPI in increasing the efficacy of standard chemotherapy and significantly improving the quality of life of treated pets, in either standard treatment [21] or metronomic regimens [22]. More recently, a metanalysis in head and neck tumor patients confirmed an increased response in patients receiving anti-acidic drugs, particularly those treated with PPI [54].

## Conclusions

These results should induce to sit down and think to new anti-tumor strategies in which PPI should be included, and highly support some commentaries and reviews published in JTM proposing inhibitors of ion and proton exchangers as a new anti-cancer approach [13, 55]. One open question might be: “How this approach may be accepted being based on a feature that is common to virtually all cancers, when the mainstream approach of research is to set up new therapies that should distinguish between cancer and cancer patients”. With this commentary we would like to propose that cancers have much more common features than peculiar molecular pathways. 
